# LED-Induced Microglial Activation and Rise in Caspase3 Suggest a Reorganization in the Retina

**DOI:** 10.3390/ijms221910418

**Published:** 2021-09-27

**Authors:** Boglárka Balogh, Gergely Szarka, Ádám J. Tengölics, Gyula Hoffmann, Béla Völgyi, Tamás Kovács-Öller

**Affiliations:** 1 János Szentágothai Research Centre, University of Pécs, 7624 Pécs, Hungary; boglarka.balogh311@gmail.com (B.B.); gergely.sz@gmail.com (G.S.); tengo.adam2@gmail.com (Á.J.T.); hgyula@gamma.ttk.pte.hu (G.H.); volgyi01@gamma.ttk.pte.hu (B.V.); 2 Retinal Electrical Synapses Research Group, National Brain Research Program (NAP 2.0), Hungarian Academy of Sciences, 1051 Budapest, Hungary; 3 Institute of Biology, Faculty of Sciences, University of Pécs, 7624 Pécs, Hungary; 4 Medical School, University of Pécs, 7624 Pécs, Hungary

**Keywords:** LED, light, retina, microglia, caspase, apoptosis, Bcl-2, BAX, degeneration

## Abstract

Vision is our primary sense as the human eye is the gateway for more than 65% of information reaching the human brain. Today’s increased exposure to different wavelengths and intensities of light from light emitting diode (LED) sources could induce retinal degeneration and accompanying neuronal cell death. Damage induced by chronic phototoxic reactions occurring in the retina accumulates over years and it has been suggested as being responsible for the etiology of many debilitating ocular conditions. In this work, we examined how LED stimulation affects vision by monitoring changes in the expression of death and survival factors as well as microglial activation in LED-induced damage (LID) of the retinal tissue. We found an LED-exposure-induced increase in the mRNA levels of major apoptosis-related markers BAX, Bcl-2, and Caspase-3 and accompanying widespread microglial and Caspase-3 activation. Everyday LED light exposure was accounted for in all the described changes in the retinal tissue of mice in this study, indicating that overuse of non-filtered direct LED light can have detrimental effects on the human retina as well.

## 1. Introduction

When Thomas Edison invented the first light bulb in 1879, the effects of light pollution were unknown, but nowadays people almost never experience total darkness. We are continuously exposed to artificial illumination, which in recent decades has become ever increasing due to recent developments in the light emitting diode (LED) industry [1,2]. As our modern society has become accustomed to the constant presence of bright light, the harmful effects of these unnatural light sources have been underestimated for the most part. Although it is known how light affects our day-and-night rhythm [3], its direct effects on the retinal tissue and corresponding functional and pathological changes have mostly been ignored. Vision is our dominant sense since more than two-thirds of the surrounding information is perceived and preprocessed by the retina. Therefore, our goal in this study is to fill this gap and explore how excess artificial light, coming from LEDs, could lead to retinal degeneration and increase the chance of retinal diseases.

Thanks to evolution, the human retina has fully adapted to natural light, but might not be ready for modern lighting solutions and the extensive use of LED screens. Compared to natural light, the light from LEDs is not homogenous in wavelength intensities and thus overrepresents lower wavelengths, less common in natural light (Figure S1).

The retina is formed by a complex network of neurons supported by glial elements in order to detect and process the information arising from the different light sources. Neurons and glia are in a fragile codependent relationship. As light passes through the layers of the retina it has the ability to affect all retinal cell types, possibly by photochemical damage, also referred to as phototoxic damage. The resulting toxic, chemically reactive molecules, such as reactive oxygen species (ROS), are the hallmarks of this adverse effect. The process occurs through light absorption of chromophores that leads to energy transfer, which in turn either chemically transforms or interacts with other molecules, resulting in secondary changes of both interacting molecules. Alternatively, this excitation energy can be propagated and transferred to further partners. The high-intensity blue spike in LEDs (400–440 nm) contains high-energy photons that eventually reach the retina, before which they must have traveled through multiple refractive tissue layers [1]. Before it reaches the photoreceptors (PRs), the site of phototransduction, this light will have to go through the layers and cells of the retina that has a cc. 150 μm thickness in total. These cells include the neuronal cell types, the ganglion cells (GCs), amacrine cells (ACs), bipolar cells (BCs), horizontal cells (HCs), and the PRs, as well as the glial cells, microglia (MG), Müller cells (MCs), and astrocytes (ACs). MGs are the most important intrinsic immune cells throughout the whole central nervous system [4,5], thus in the retina itself [6].

Although there have been many publications involving high-intensity light damage in the retina [7,8,9], and the effects of artificial light on the circadian rhythms [10,11,12,13,14], everyday intensities of artificial light levels (500–2000 lux, [15]) are rarely studied [15,16]. The long-term effects have no related literature at all. However, we have data regarding that; the intensity of the light has an obvious impact on the action potential frequencies of the RGCs and thus on visual coding [17].

The key proteins involved in retinal apoptosis, which have also been established in order to study widespread damage in the retina, are BAX, Bcl-2, and Casp3 [18,19,20,21] but some milder and earlier effects of degeneration can be detected by microglial activation in the retina [22,23], taking into account that this cell type acts as the primary immune cells in the retina, indicating any kind of harmful event [24].

Our work helps us to understand how our modern lifestyle can partake in vision-debilitating diseases, such as age-related macular degeneration (AMD), which has been shown to have a higher incidence rate in advanced countries [25,26], where the use of modern LED technologies is also more widespread [27,28].

## 2. Results

### 2.1. Changes in the Expression of Cell Death Factors Show Possible Retinal Reorganization

**Figure 1:**
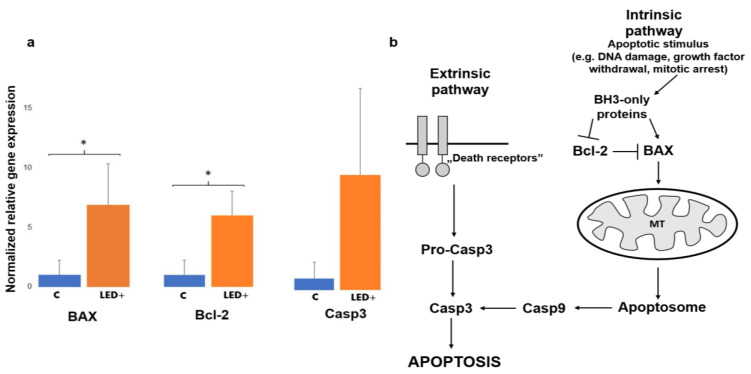
Bax, Bcl-2, and Casp3 gene expression in the retina of control and LED-treated mice. (**a**) Histograms show the relative mRNA expression of Bax, Bcl-2, and Casp3 as measured in control (normalized to 1) and LED-treated mice (*n* = 8, 4 control, 4 LED +). (* *p* = 0.05; *p*_Casp3_ = 0.1). (**b**) The main players of apoptosis. Casp3 is the link between intrinsic and extrinsic pathways, while Bcl-2 has a role in survival.

In order to determine whether LED exposure has detrimental effects on the mouse retina, we compared the aforementioned three well-known markers (Bax, Bcl-2, and Casp3) involved in cell death signaling between the LED-exposed and our control retinas. We saw a substantial increase in the expression of all three of these markers. Our results show that the levels of Bax and Bcl-2 statistically significantly increased in LED-treated animals (6.89-fold, and 6.14-fold). The Casp3 mRNA level increase (10.20-fold) did not appear to be statistically significantly different, due to the large variation between samples (Figure 1).

### 2.2. Microglia Activation in the Superficial Layer

**Figure 2:**
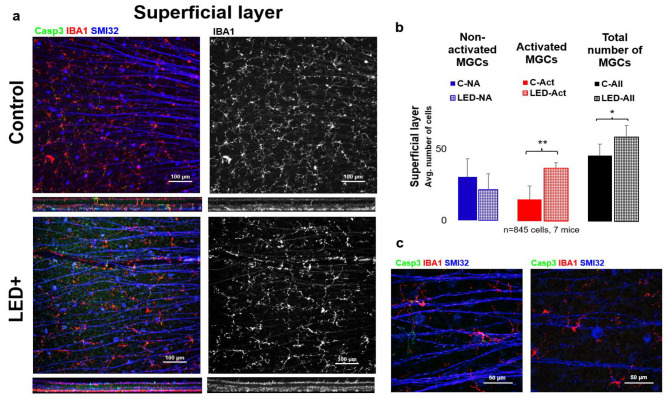
Microglia activation in the superficial layer (inner retina GCL). Panel (**a**) shows the ganglion cell layer of control and LED retinas labeled SMI32 (GC marker), Iba1 (MG marker), and Casp3 (Casp3+ cells). The colors shown are the same as the colors above the images. In the second column, only Iba1-labeled microglia are shown in white. In panel (**b**) the histogram shows the number of non-activated, activated, and total microglia counted in the SL (* *p* = 0.05; ** *p* = 0.01). (**c**) Shows high-magnification confocal microscopic images scanned with a 63× objective of activated MGs (left) and non-activated MGs (right).

As a result of LED treatment and the primary inflammatory processes, microglia activation significantly increased based on our cell counts (*n* = 845 cells in 7 retinas) in the superficial layer of the retina. In addition, we observed that not only did the number of activated microglia increase with LED light treatment, but we also noted a significant increase in the total number of microglia (Figure 2).

**Figure 3:**
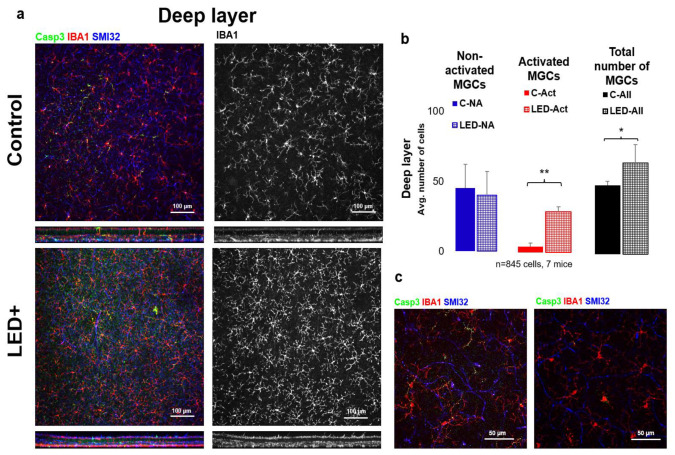
Microglia activation in the deeper parts of the retina. Panel (**a**) of the figure shows the outer or “deep” layer of control and LED retinas, labeled with SMI32 (neuron marker), Iba1 (microglia marker), and Casp3 (Casp3 + cells) scanned with a 20× objective (pictured). The colors shown are the same as the colors above the images. In the second column, only Iba1-labeled microglia are shown in white. In (**b**) the histogram shows the number of inactivated, activated, and total microglia counted in the retinal ganglion cell layer and inner fibrous layer (* *p* = 0.05; ** *p* = 0.01). (**c**) Shows confocal microscopic images scanned with a 63× objective of activated MGs (left) and non-activated MGs (right).

In the LED-treated animals, a 6.4-fold (from 5 to 32 in avg.) increase was observed in the number of activated MGs, accompanied by a rise in total MG count (from 55 to 72 in avg.) (*n* = 845 cells in 7 retinas) in the deeper layers of the retina (INL to ONL) (Figure 3).

### 2.3. Rise in Casp3 Activation as a Result of LED Treatment

**Figure 4:**
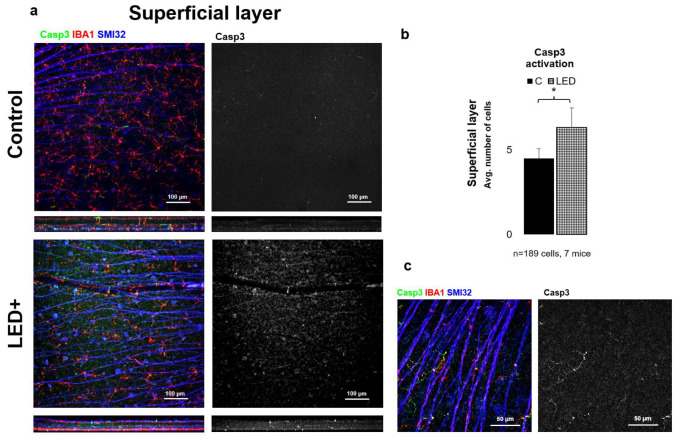
Casp3 activation in the superficial layer. Panel (**a**) shows the ganglion cell layer of control and LED retinas labeled SMI32 (GC marker), Iba1 (MG marker), and Casp3 (Casp3+ cells) scanned with a 20× objective in a confocal microscope. The colors shown are the same as the colors above the images. In the second column, only Casp3-labeled cells are shown in white. In panel (**b**) the histogram shows the number of Casp3-activated cells in the SL (* *p* = 0.05). Panel (**c**) shows high-magnification confocal microscopic images scanned with 63× objective of Casp3+ areas in MG.

**Figure 5:**
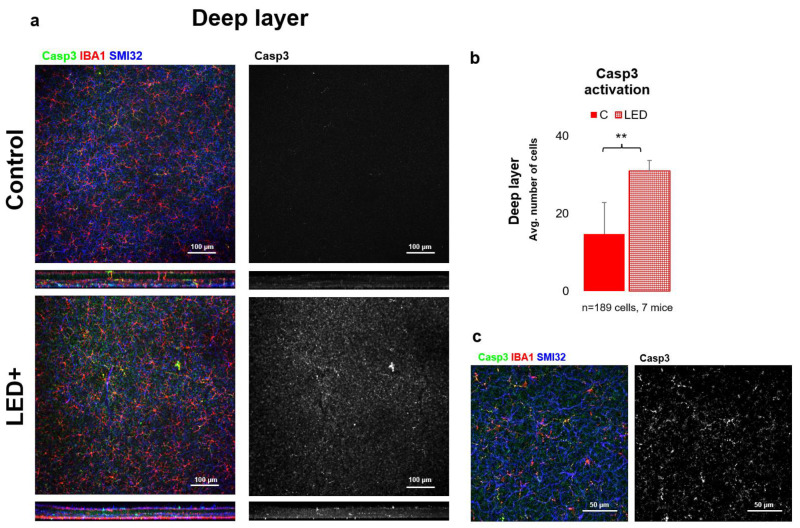
Casp3 activation in the deeper parts of the retina. Panel (**a**) of the figure shows the outer core layer of control and LED retinas labeled SMI32 (GC marker), Iba1 (MG marker), and Casp3 (Casp3+ cells) scanned with a 20× objective in a confocal microscope. The colors shown are the same as the colors above the images. In the second column, only Casp3-activated cells are shown in white. In panel (**b**) the histogram shows the number of activated cells counted in the DL (** *p* = 0.01). Panel (**c**) shows high-magnification confocal microscopic images scanned with a 63× objective. The triple-labeled and only Casp3-activated cells are visible from the LED retinas.

To gain a more detailed picture of the changes occurring in the retina due to LED treatment, we performed immunohistochemical experiments for Casp3 expression. Based on our results, we observed that in the superficial layer mainly neuronal cells (mostly GCs) became Casp3 positive, while in the deep layer MGs showed Casp3 positivity. After cell counting (*n* = 189 cells in 7 mice), we can also see a significant increase in the total number of Casp3+ cells in both the superficial and deep layers; however, the degree of activation was lower in the superficial layer (Figure 4) compared to the deep layer (Figure 5).

## 3. Discussion

### 3.1. Possible Effects of LED Light in the Retina

Based on the relatively sparse information available on the effects of light sources, such as LED illumination, it can be seen that light damage can manifest varied effects in the retina [29]. Most studies link phototoxic damage to the photoreceptors and retinal pigment epithelium (RPE), where lipofuscin-containing phototoxic compounds tend to accumulate. Marie and colleagues have shown that the cone inner segments can sustain phototoxic damage due to visible violet light (~430 nm) exciting porphyrin compounds [30]. Taking this hypothesis into account, we studied LED exposure effects on the whole retinal unit. Since the inner retina is central in signal processing and output, it is crucial to determine whether inner retinal cells and connections can also be affected the same way. Thus, we identified the key players in retinal damage and possible reorganization and tested how their expressional patterns change in mice due to exposure to LED light equivalent to the amount an average office worker is exposed to in their everyday life. However, in our experimental setup the LED+ retinas were compared to a low level of fluorescent light, not containing a high-intensity blue pattern; it is still clear that all the key players tested showed a major increase in the LED+ retinas, thus suggesting a reorganization.

### 3.2. The Activation of BAX, Bcl-2, and Casp3 Shows Possible Reorganization in the Retina

One of the main goals of this study was to compare the expression levels of some of the central proteins involved in apoptosis and cellular survival after a normal level of LED light exposure. We detected a major increase in the expression of two key pro-apoptotic proteins, BAX and Casp3. BAX is involved in the death-signal transmission towards the mitochondria, while activated Casp3 is a major executioner protein merging apoptotic signals from the extrinsic and intrinsic apoptotic pathways [31]. Our results show that the levels of Bax and Bcl-2 increased statistically significantly in LED-treated animals, while the levels of Casp3 showed an indicative increase that was not statistically significant due to the high variance between the samples (Figure 1). In comparison, however, based on the immunohistological experiments performed to identify Casp3-expressing cells, we saw a statistically significant increase in overall Casp3 expression. We showed (Figure 5) that Casp3 expression increases in GCs in the superficial retinal layer and microglia in the deeper retinal layer, and based on these results, it can be concluded that the apoptotic change is likely to be balanced by the anti-apoptotic effect of Bcl-2 production. However, it should be noted that this procedure is not sufficient to detect actual cell death, but it is a good indicator of the trends, as the microglial activation is often associated with Casp3 activation without the adverse effects of actual cell death. This comes from their ability to escape the Casp3 effector function [32,33]. When we compare the general picture gained from the qPCR results, where we saw a statistically insignificant increase in Casp3 levels, we can see that the number of Casp3-expressing cells indeed shows a significant increase that is more prominent in the deep layer of the retina.

### 3.3. Microglial Activation as a Hallmark of Retinal Inflammation

Microglia in the retina are specialized macrophages responsible for immune function and they are part of the three retinal glial cell types together with astrocytes and Müller cells; from previous reports we can see that microglia activation state, based on morphological features, is a precise and early detection method to determine retinal damage [24]. Therefore, we used the microglia, the primary, intrinsic immune cells in the retina, as sensors for retinal integrity. Any loss of integrity can be detected by microglial activation. The change to arise from activated morphology is well-visible on the cells after treatment, as their somata become enlarged and the primary protrusions are thickened, while some more activated forms show even more amoeboid-like morphologies [34,35].

Our results show microglial activation both superficially and in the deep layer of microglia. The morphological activation is also coupled with Casp3 activation in the case of microglia.

Interestingly the rise in the number of activated microglial cells is combined with a small surge in the number of microglia. This could come from either the infiltrating macrophages or the division of intrinsic microglia, as they both express microglia/macrophage-specific calcium-binding protein (IBA1). Studying the morphological features of the IBA1+ cells, we could not identify infiltrating monocytes/macrophages, thus leading to the conclusion that the rise in IBA1+ cells is due to the division of the retina’s local microglial populations, resulting in a significant increase in the total number of microglia (Figure 2 and Figure 3).

### 3.4. How to Avoid Retinal Damage Caused by LEDs?

The effects shown by us here should be taken as a warning sign and they warrant further research to be performed. It is likely that in the future the LED technologies will flourish even further, leading to an even greater increase in LED use, which the vertebrate eye is not likely to withstand without damage to its integrity. Our results indicate that filtering high-energy blue light could reduce potential harm in the retina, which can be conducted using filtering spectacles when exposed to LED lights for prolonged periods of time [36]; alternatively, LED manufacturers should consider producing LEDs with different spectral features and make further efforts to avoid eye damage in the long term.

## 4. Materials and Methods

### 4.1. Animals and Preparation

Animal handling, housing, and experimental procedures were reviewed and approved by the ethical committee of the University of Pécs (BA02/2000-6/2006). All animals were treated in accordance with the ARVO Statement for the Use of Animals in Ophthalmic and Vision Research. All efforts were made to minimize pain and discomfort during the experiments. Mice were deeply anesthetized with the inhalation of Forane (4%, 0.2 mL/L) and then sacrificed using cervical dislocation. Eyes of mice (Mus musculus, C57BL/6J, 1–12 months, *n* = 10 animals, all males) were immediately removed after termination. Eyeballs were cut at the ora serrata, and lens and vitreous body were removed. Retinas were fixed in 4% PFA in 1× PBS at room temperature for 15 min (for details, see: [37]).

### 4.2. LED Treatment

The LED treatment of mice was performed with an LED box that is basically a ring of 4 chip on board (COB) LEDs (22 mm, 3 W, neutral white, ~4500 K) in parallel with the box’s wall, surrounding the transparent cage with a warm-light LED bulb (Tungsram E27, 8 W, ~3000 K) hanging over the cage. The summated, measured intensity of the LED light was 1000 lux (see the wavelength-intensity profile in Figure S1) in the center of the cage, which is equivalent to a brightly lit office [38,39]. The animals were held under this light for 7 days, 14 h a day, and provided with food and water ad libitum with a cage change on the third day.

### 4.3. Quantitative PCR Analysis

One of the retinae of LED+ and control mice (*n* = 4/4) was fast-frozen in nuclease-free 1.5 mL tubes by liquid nitrogen immersion and stored at −80 °C until processed with RNasol RT [40]. The resulting RNA (normalized to 1 µg) was reverse transcribed with oligo-dT primer to cDNA with RevertAid First Strand cDNA Synthesis Kit (Thermo Fisher, Waltham, MA, USA), using a MiniPCR (miniPCRbio, Cambridge, MA, USA) with the following steps: 10 min, 25 °C; 60 min, 42 °C; 10 min, 70 °C. The resulting cDNA was then stored at −20 °C until use [41].

We used 20 nanograms of the resulting cDNA in each of the wells, in triplicates for each sample, to perform the expression analysis for BAX, Bcl-2, and Casp3, with the use of RPL13a endogenous control (see the primers in Table 1) with an SYBR-green master mix [42].

We used the following protocol in a BioRad CFX Connect (BioRad, Hercules, CA, USA [41]) for the amplification and detection of fluorescent intensities in the samples: 5 min, 95 °C; 20 s at 93 °C; 20 s, 54 °C; 30 s at 72 °C; 39× repeat, 2 min at 72 °C. A melt curve analysis was performed afterward. The ΔCTs were analyzed with CFX Connect software and MS Excel.

### 4.4. Immunohistochemistry and Microscopy

Following a thorough washing of the fixed eyecups in PBS, the retinas were isolated. Blocking was performed with bovine serum albumin 5%, Triton X-100 0.5%, and Na-azide 0.05% in PBS (BTA) on the bottom of a 24-well plate. Primary antibodies were used as indicated in Table 2. After washing (×3) with PBS, secondary antibodies were added (Table 2). Retinas were mounted with VectaShield (Vector Labs., Burlingame, CA, USA), using nr.1 cover slides, and inspected using a Zeiss LSM 710 confocal laser-scanning microscope (Plan Apochromat 20× and 63× objectives; NA: 1.4; Carl Zeiss Inc., Jena, Germany) with normalized laser power and filter settings. We made 1.5 and 0.5 μm thin optical sections with the 20× and 63× objectives (details in [43]).

### 4.5. Measurement of Microglial Activation

All measurements were performed using FIJI (NIH, Bethesda, MD, USA [44]). First, we performed two z-merges from the 5-5 stacks (3.75 µm) for the superficial and deep regions of MGs using only mid-central retinal scans. Cells were manually grouped one by one according to their morphologies into activated and non-activated, using the “Cell-counter” plugin in FIJI, according to the morphological classifications of Lawson and colleagues and others [34,35,45]. Only cells with the whole visible area were included; we omitted the ones on the edges.

### 4.6. Statistical Analyses

One-way ANOVA analyses were performed using the Origin18 (Origin, Version 2018b, OriginLab Corporation, Northampton, MA, USA). Normal distribution was previously confirmed through statistical analysis.

## Figures and Tables

**Table 1:** Primers.

Primer Name	Sequence
BAX forward	5′ GGT CTT CTT CCG GGT GGC AG 3′
BAX reverse	5′ CTT CCC AGC CAC CCT GGT CTT G 3′
Bcl-2 forward	5′ ACC TGA ACC GGC ATC TGC AC 3′
Bcl-2 reverse	5′ CTT GTG GCC CAG GTA TGC ACC 3′
Casp3 forward	5′ CGG GGA GCT TGG AAC GGT ACG 3′
Casp3 reverse	5′ TCC CAG AGT CCA CTG ACT TGC T 3′
RPL13 forward	5′ CCA GAG GTT TTG GGG TCA GAA 3′
RPL13 reverse	5′ GCA GTT GCA GAC AAA CTG GAG G 3′

**Table 2:** Antibodies.

Primary Antibodies				Secondary Antibodies, Dyes			
Name	Dilution	Source	Code	Name	Dilution	Source	Code
ms-SMI32	1:1000	Calbiochem	NE1023	anti-ms-Alexa488	1:1000	Invitrogen	A11017
gp-Iba1	1:2000	SySy	234004	anti-gp-Alexa647	1:1000	Invitrogen	A21450
rb-Caspase-3	1:1000	NovusBio	AF835	anti-rb-Cy3	1:500	Jackson	715-165-150

## Data Availability

All data are available from the authors upon reasonable request.

## Supplementary Materials

The following are available online at https://www.mdpi.com/article/10.3390/ijms221910418/s1, Figure S1: The short wavelength spike of the LEDs used in our study; (a) the operation principle of the Theremino-based spectrophotometer; (b) the wavelength-intensity profile of sunlight; (c) the wavelength-intensity profile of the LEDs used in our study, with the short blue wavelength (443 nm) spike indicated with a red arrow.

## References

[1] Behar-Cohen F., Martinsons C., Viénot F., Zissis G., Barlier-Salsi A., Cesarini J.P., Enouf O., Garcia M., Picaud S., Attia D. (2011). Light-emitting diodes (LED) for domestic lighting: Any risks for the eye?. Prog. Retin. Eye Res..

[2] Pust P., Schmidt P.J., Schnick W. (2015). A revolution in lighting. Nat. Mater..

[3] Dauchy R.T., Blask D.E., Hoffman A.E., Xiang S., Hanifin J.P., Warfield B., Brainard G.C., Anbalagan M., Dupepe L.M., Dobek G.L. (2019). Influence of Daytime LED Light Exposure on Circadian Regulatory Dynamics of Metabolism and Physiology in Mice. Comp. Med..

[4] Winkler Z., Kuti D., Polyák Á., Juhász B., Gulyás K., Lénárt N., Dénes Á., Ferenczi S., Kovács K.J. (2019). Hypoglycemia-activated Hypothalamic Microglia Impairs Glucose Counterregulatory Responses. Sci. Rep..

[5] Császár E., Lénárt N., Cserép C., Környei Z., Fekete R., Pósfai B., Balázsfi D., Hangya B., Schwarcz A.D., Szöllősi D. (2021). Microglia control cerebral blood flow and neurovascular coupling via P2Y12R-mediated actions. BioRxiv.

[6] Okunuki Y., Mukai R., Nakao T., Tabor S.J., Butovsky O., Dana R., Ksander B.R., Connor K.M. (2019). Retinal microglia initiate neuroinflammation in ocular autoimmunity. Proc. Natl. Acad. Sci. USA.

[7] Pollak V.A., Romanchuk K.G. (1980). The risk of retina damage from high intensity light sources. Am. Ind. Hyg. Assoc. J..

[8] Jaadane I., Boulenguez P., Chahory S., Carré S., Savoldelli M., Jonet L., Behar-Cohen F., Martinsons C., Torriglia A. (2015). Retinal damage induced by commercial light emitting diodes (LEDs). Free Radic. Biol. Med..

[9] Vicente-Tejedor J., Marchena M., Ramírez L., García-Ayuso D., Gómez-Vicente V., Sánchez-Ramos C., de la Villa P., Germain F. (2018). Removal of the blue component of light significantly decreases retinal damage after high intensity exposure. PLoS ONE.

[10] Cho Y., Ryu S.H., Lee B.R., Kim K.H., Lee E., Choi J. (2015). Effects of artificial light at night on human health: A literature review of observational and experimental studies applied to exposure assessment. Chronobiol. Int..

[11] Touitou Y., Reinberg A., Touitou D. (2017). Association between light at night, melatonin secretion, sleep deprivation, and the internal clock: Health impacts and mechanisms of circadian disruption. Life Sci..

[12] Lunn R.M., Blask D.E., Coogan A.N., Figueiro M.G., Gorman M.R., Hall J.E., Hansen J., Nelson R.J., Panda S., Smolensky M.H. (2017). Health consequences of electric lighting practices in the modern world: A report on the National Toxicology Program’s workshop on shift work at night, artificial light at night, and circadian disruption. Sci. Total Environ..

[13] Aulsebrook A.E., Jones T.M., Mulder R.A., Lesku J.A. (2018). Impacts of artificial light at night on sleep: A review and prospectus. J. Exp. Zool. Part A Ecol. Integr. Physiol..

[14] Tähkämö L., Partonen T., Pesonen A.K. (2019). Systematic review of light exposure impact on human circadian rhythm. Chronobiol. Int..

[15] Cole R.J., Kripke D.F., Wisbey J., Mason W.J., Gruen W., Hauri P.J., Juarez S. (1995). Seasonal variation in human illumination exposure at two different latitudes. J. Biol. Rhythm..

[16] Potter G.D., Skene D.J., Arendt J., Cade J.E., Grant P.J., Hardie L.J. (2016). Circadian Rhythm and Sleep Disruption: Causes, Metabolic Consequences, and Countermeasures. Endocr. Rev..

[17] Tengölics Á.J., Szarka G., Ganczer A., Szabó-Melegh E., Nyitrai M., Kovács-Öller T., Völgyi B. (2019). Response Latency Tuning by Retinal Circuits Modulates Signal Efficiency. Sci. Rep..

[18] Kluck R.M., Bossy-Wetzel E., Green D.R., Newmeyer D.D. (1997). The release of cytochrome c from mitochondria: A primary site for Bcl-2 regulation of apoptosis. Science.

[19] Kroemer G., Dallaporta B., Resche-Rigon M. (1998). The mitochondrial death/life regulator in apoptosis and necrosis. Annu. Rev. Physiol..

[20] Thornberry N.A., Lazebnik Y. (1998). Caspases: Enemies within. Science.

[21] Su L.J., Zhang J.H., Gomez H., Murugan R., Hong X., Xu D., Jiang F., Peng Z.Y. (2019). Reactive Oxygen Species-Induced Lipid Peroxidation in Apoptosis, Autophagy, and Ferroptosis. Oxidative Med. Cell. Longev..

[22] Silverman S.M., Wong W.T. (2018). Microglia in the Retina: Roles in Development, Maturity, and Disease. Annu. Rev. Vis. Sci..

[23] Kinuthia U.M., Wolf A., Langmann T. (2020). Microglia and Inflammatory Responses in Diabetic Retinopathy. Front. Immunol..

[24] Rashid K., Akhtar-Schaefer I., Langmann T. (2019). Microglia in Retinal Degeneration. Front. Immunol..

[25] Flaxman S.R., Bourne R., Resnikoff S., Ackland P., Braithwaite T., Cicinelli M.V., Das A., Jonas J.B., Keeffe J., Kempen J.H. (2017). Global causes of blindness and distance vision impairment 1990–2020: A systematic review and meta-analysis. Lancet Glob. Health.

[26] Ambati J., Ambati B.K., Yoo S.H., Ianchulev S., Adamis A.P. (2003). Age-related macular degeneration: Etiology, pathogenesis, and therapeutic strategies. Surv. Ophthalmol..

[27] Hyman L., Neborsky R. (2002). Risk factors for age-related macular degeneration: An update. Curr. Opin. Ophthalmol..

[28] National Eye Institute (2010). Eye Health Data and Statistics. Age-Related Macular Degeneration (AMD) Data and Statistics. https://www.nei.nih.gov/learn-about-eye-health/outreach-campaigns-and-resources/eye-health-data-and-statistics/age-related-macular-degeneration-amd-data-and-statistics.

[29] Youssef P.N., Sheibani N., Albert D.M. (2011). Retinal light toxicity. Eye.

[30] Marie M., Forster V., Fouquet S., Berto P., Barrau C., Ehrismann C., Sahel J.A., Tessier G., Picaud S. (2020). Phototoxic damage to cone photoreceptors can be independent of the visual pigment: The porphyrin hypothesis. Cell Death Dis..

[31] Fricker M., Tolkovsky A.M., Borutaite V., Coleman M., Brown G.C. (2018). Neuronal Cell Death. Physiol. Rev..

[32] Burguillos M.A., Deierborg T., Kavanagh E., Persson A., Hajji N., Garcia-Quintanilla A., Cano J., Brundin P., Englund E., Venero J.L. (2011). Caspase signalling controls microglia activation and neurotoxicity. Nature.

[33] Kavanagh E., Rodhe J., Burguillos M.A., Venero J.L., Joseph B. (2014). Regulation of caspase-3 processing by cIAP2 controls the switch between pro-inflammatory activation and cell death in microglia. Cell Death Dis..

[34] Lawson L.J., Perry V.H., Dri P., Gordon S. (1990). Heterogeneity in the distribution and morphology of microglia in the normal adult mouse brain. Neuroscience.

[35] Streit W.J., Walter S.A., Pennell N.A. (1999). Reactive microgliosis. Prog. Neurobiol..

[36] Leung T.W., Li R.W., Kee C.S. (2017). Blue-Light Filtering Spectacle Lenses: Optical and Clinical Performances. PLoS ONE.

[37] Völgyi B., Abrams J., Paul D.L., Bloomfield S.A. (2005). Morphology and tracer coupling pattern of alpha ganglion cells in the mouse retina. J. Comp. Neurol..

[38] Heil D.P., Mathis S.R. (2002). Characterizing free-living light exposure using a wrist-worn light monitor. Appl. Ergon..

[39] Crowley S.J., Molina T.A., Burgess H.J. (2015). A week in the life of full-time office workers: Work day and weekend light exposure in summer and winter. Appl. Ergon..

[40] RNAzol^®^ RT RNA Isolation Reagent User Manual. https://res.mdpi.com/data/mdpi_references_guide_v5.pdf.

[41] Kovács-Öller T., Raics K., Orbán J., Nyitrai M., Völgyi B. (2014). Developmental changes in the expression level of connexin36 in the rat retina. Cell Tissue Res..

[42] SYBR^®^ Green PCR Master Mix and SYBR^®^ Green RT-PCR Reagents Kit User Manual. https://www.thermofisher.com/document-connect/document-connect.html?url=https%3A%2F%2Fassets.thermofisher.com%2FTFS-Assets%2FLSG%2Fmanuals%2Fcms_041053.pdf.

[43] Kovács-Öller T., Szarka G., Tengölics Á.J., Ganczer A., Balogh B., Szabó-Meleg E., Nyitrai M., Völgyi B. (2020). Spatial Expression Pattern of the Major Ca^2+^-Buffer Proteins in Mouse Retinal Ganglion Cells. Cells.

[44] Schindelin J., Arganda-Carreras I., Frise E., Kaynig V., Longair M., Pietzsch T., Preibisch S., Rueden C., Saalfeld S., Schmid B. (2012). Fiji: An open-source platform for biological-image analysis. Nat. Methods.

[45] Davis E.J., Foster T.D., Thomas W.E. (1994). Cellular forms and functions of brain microglia. Brain Res. Bull..

