# Supplementary figures and images for: LED-Induced Microglial Activation and Rise in Caspase3 Suggest a Reorganization in the Retina
Source: Int J Mol Sci. 2021 Sep 27;22(19):10418. doi: 10.3390/ijms221910418 (PMC8508983; doi:10.3390/ijms221910418)

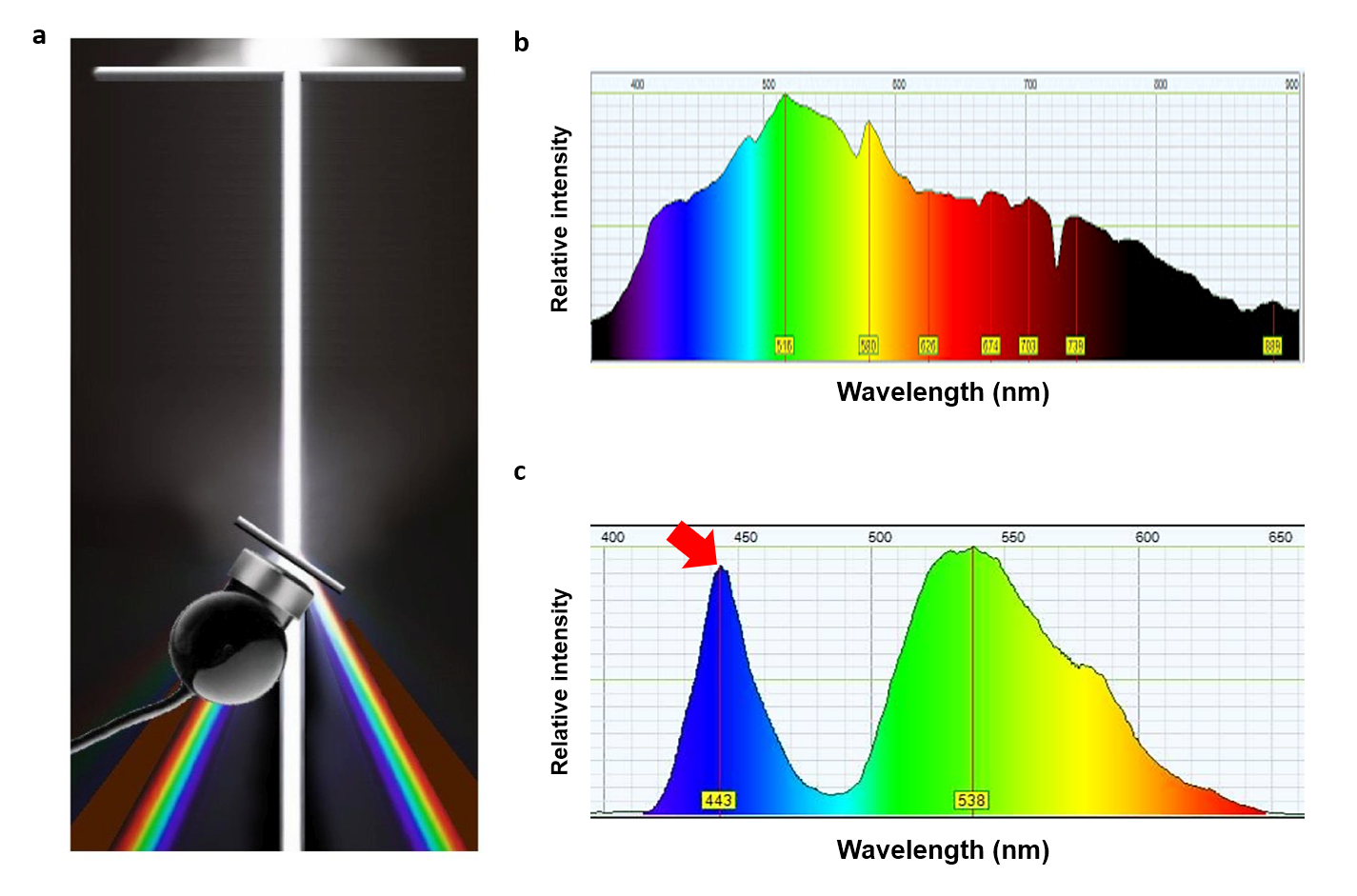

Supplement: Supplementary file 1 [file ijms-22-10418-s001.zip › ijms-1351100-supplementary.tif]
